# Economic burden of lives lost due to COVID-19 in New York State

**DOI:** 10.2217/cer-2021-0086

**Published:** 2021-06-01

**Authors:** Briana Lui, Michelle Zheng, Robert S White, Marguerite Hoyler

**Affiliations:** ^1^Department of Anesthesiology, Weill Cornell Medicine, 525 East 68th Street, Box 124, New York, NY 10065, USA; ^2^College of Human Ecology, Cornell University, Martha Van Rensselaer Hall, Ithaca, NY 14850, USA

**Keywords:** COVID-19, health economics, New York State, value of statistical life, years of potential life lost

## Abstract

**Aim:** To examine the economic impact of lives lost due to the COVID-19 pandemic across New York State. **Materials & methods:** Death counts by age range and period life expectancy were extracted from the NYS Department of Health, NYC Department of Health and Mental Hygiene, and Social Security Administration website. Years of potential life lost and value of statistical life (VSL) were calculated. **Results:** The average years of potential life lost per person was 12.72 and 15.13, and the VSL was US$119.62 and 90.45 billion, in NYS and NYC, respectively. VSL was greatest in Queens and Brooklyn, followed by the Bronx, Manhattan and Staten Island. **Conclusion:** New York City, specifically Queens and Brooklyn, bore the greatest economic burden of lives lost across the state.

The loss of life due to the COVID-19 pandemic has been devastating, with over 2.6 million deaths worldwide and over 500,000 deaths in the USA alone as of March 2021 [1]. This staggering number of premature deaths imposes a substantial economic burden on society. As the death toll continues to rise, it is crucial that we understand the economic value of these lives lost to guide policymaking decisions at a national, state and local level. Some efforts have been made to estimate the fiscal burden of mortality early on in the pandemic, including previous work examining the expected loss of life-years and costs associated with premature deaths in the state of Ohio [2,3]. These studies incorporated the value of a statistical life year (VSLY) and value of a statistical life (VSL) – concepts frequently used in economic and public policy analyses to estimate the financial impact of premature mortality, and to inform cost–benefit interventions for reducing mortality risk – to estimate the monetary costs of death due to COVID-19. However, much work is still needed to fully understand the economic impact of lives lost due to COVID-19.

New York City was the early epicenter of the pandemic in the USA, reporting approximately 203,000 cases of COVID-19 in just the first 3 months of the pandemic and a crude fatality rate of 9.2% among confirmed cases and 32.1% among hospitalized patients [4]. To date, COVID-19 has contributed to 39,093 deaths in New York State, of which the majority is concentrated in New York City. This study aims to expand upon prior work to estimate the years of potential life lost (YPLL) and the statistical value of these lives lost due to COVID-19 in New York State and New York City as well as the economic burden of lives lost in the five boroughs: the Bronx, Brooklyn, Manhattan, Queens and Staten Island.

## Materials & methods

### Data acquisition

State, city and borough-level data on cumulative COVID-19 deaths were extracted from the New York State Department of Health website (https://covid19tracker.health.ny.gov/views/NYS-COVID19-Tracker/NYSDOHCOVID-19Tracker-Fatalities?%3Aembed=yes&%3Atoolbar=no&%3Atabs=n) [5], New York City Department of Health and Mental Hygiene website (https://www1.nyc.gov/site/doh/covid/covid-19-data.page) [6], and GitHub repository (https://github.com/nychealth/coronavirus-data) on 8 March 2021 [7]. Data included all known COVID-19 death counts by age range in New York State (inclusive of New York City) and New York City (further stratified by borough: the Bronx, Brooklyn, Manhattan, Queens and Staten Island). Data are updated daily at a 1-day lag for New York State and a 3-day lag for New York City, and are subject to change as fatality reports are confirmed and validated, reporting dates back to 1 March 2020 and 29 February 2020 for New York State and New York City, respectively.

The most recent 2017 actuarial life tables for the Social Security area population were obtained from the Social Security Administration website to determine the number of years of life remaining at a given age [8]. Acquired data included period life expectancy (defined as the average number of years of life remaining if a group of persons at that age were to experience the mortality rates for 2017 over the course of their remaining life) for males and females from birth to 119 years of age. The Social Security area population includes residents of the 50 States and the District of Columbia (adjusted for net census undercount); civilian residents of Puerto Rico, the Virgin Islands, Guam, American Samoa and the Northern Mariana Islands; Federal civilian employees and persons in the US Armed Forces abroad and their dependents; noncitizens living abroad who are insured for Social Security benefits; and all other US citizens abroad. This study used publicly available data and did not require approval from the Institutional Review Board.

### Statistical analysis

The baseline age of death was determined by taking the upper value of the midpoint of each age range (e.g., 5 for age range 0–9). The age ranges for New York State were: 0–9, 10–19, 20–29, 30–39, 40–49, 50–59, 60–69, 70–79, 80–89 and 90 and over. For 90 and over, the age range was arbitrarily set to 90–99 and the midpoint was set to 95. Nine deaths of unknown age range were excluded from the New York State analysis. The age ranges for New York City (and the five boroughs) were: 0–17, 18–24, 25–34, 35–44, 45–54, 55–64, 65–74 and 75 and over. For 75 and over, the age range was set to 75–95 and the midpoint was set to 81 based on the 2018 mean life expectancy in New York City as reported by the New York City Department of Health and Mental Hygiene’s Bureau of Vital Statistics [9].

The primary outcomes of this study were total YPLL and VSL due to COVID-19 in New York State, New York City and the five boroughs. YPLL for each age range was calculated by multiplying the number of deaths by the average period life expectancy for males and females at the midpoint of each respective age range. The population average VSLY, which is used by policymakers to calculate the economic value of reducing mortality risks, was determined to be US$240,676 based on previously published literature [10]. The VSL for each age range was then calculated by multiplying the YPLL by the VSLY to quantify the economic burden of premature deaths due to COVID-19 [3]. The YPLL and VSL values were aggregated to obtain total YPLL and total VSL. Average YPLL per person was calculated by dividing the total YPLL by the number of deaths. Sensitivity analyses for New York State and New York City were performed using the upper and lower end of each age range to determine the estimated range of YPLL.

## Results

**Figure 1.**
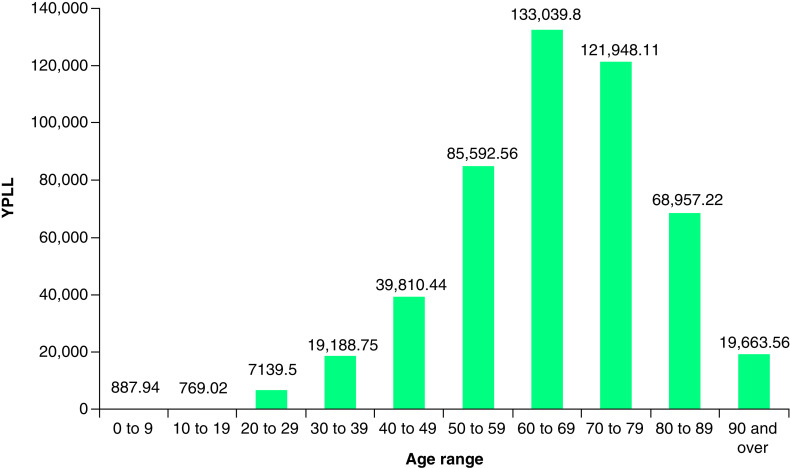
YPLL by age range for New York State. YPLL: Years of potential life lost.

As of 8 March 2021, there were 39,084 deaths due to COVID-19 across New York State. The number of lives lost at the state level resulted in a total YPLL of 496,998 (range: 412,722–599,326) and a total VSL of US$119.62 billion (range: US$99.33–144.24 billion; Table 1). The average YPLL was 12.72 per person (range: 10.56–15.33). Deaths in the 60–69 and 70–79 age ranges contributed to more than a half of the total YPLL and those in the 50–59 and 80–89 age ranges contributed to 17 and 14% of the total YPLL, respectively (Figure 1).

**Table 1.** Total YPLL & VSL for New York State and New York City.

Deaths	New York State			New York City		
	39,084			24,841		
	Total YPLL	Average YPLL (per person)	Total VSL (USD)	Total YPLL	Average YPLL (per person)	Total VSL (USD)
Base case	496,998	12.72	US$119,615,225,904	375,815	15.13	US$90,449,564,297
Lower bound	412,722	10.56	US$99,332,394,393	270,256	10.88	US$65,044,195,632
Upper bound	599,326	15.33	US$144,243,428,901	467,931	18.84	US$112,619,793,847

VSL: Value of statistical life; YPLL: Years of potential life lost.

**Figure 2.**
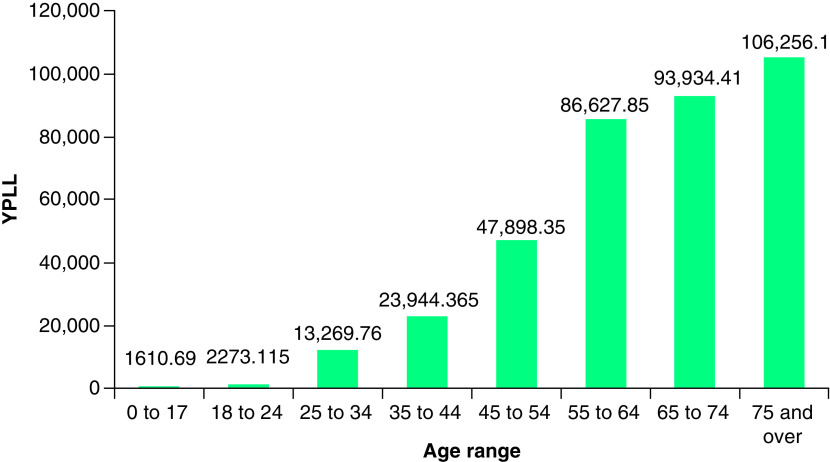
YPLL by age range for New York City. YPLL: Years of potential life lost.

In New York City, 24,841 COVID-19 deaths resulted in a total YPLL of 375,815 (range: 270,256–467,931) which corresponded to a total VSL of US$90.45 billion (range: US$65.04–112.6 billion). The average YPLL at the city level was 15.13 per person (range: 10.88–18.84). Deaths in the 55–64, 65–74 and 75 and over age ranges made up 76% of the total YPLL (Figure 2). Stratifying by borough, total YPLL and VSL were greatest in Queens and Brooklyn, followed by the Bronx, Manhattan and Staten Island (Table 2). Average YPLL per person was highest in the Bronx, followed by Queens, Brooklyn, Staten Island and Manhattan.

**Table 2.** Total YPLL & VSL stratified by borough for New York City.

Borough	Deaths	Total YPLL	Average YPLL (per person)	Total VSL (USD)
Bronx	5004	80,056	16.00	US$19,267,544,61
Brooklyn	7504	112,570	15.00	US$27,092,977,946
Manhattan	3298	44,283	13.43	US$10,657,905,850
Queens	7632	118,600	15.54	US$28,544,119,448
Staten Island	1403	20,305	14.47	US$4,887,016,433

VSL: Value of statistical life; YPLL: Years of potential life lost.

## Discussion

This study examined the YPLL and VSL due to COVID-19 in New York State, New York City and the five boroughs over a period of approximately 1 year since the start of the pandemic. As of 8 March 2021, the economic burden of lives lost was US$119.62 billion in New York State, with New York City accounting for an overwhelming majority of the cost at US$90.45 billion. The average YPLL per person was also greater in New York City compared with the overall state (15.13 vs 12.72). These findings are likely a reflection of a combination of factors including urban density, hospital bed and equipment shortages, racial/ethnic and socioeconomic disparities, local policymaker response, and New York City’s status as a major center for global trade and tourism, and an early epicenter of the pandemic in the USA [11–13]. Further, state- and city-level analysis demonstrated that the economic burden of COVID-19 was predominantly concentrated in older age groups, consistent with prior literature showing the largest increase in mortality risk in patients over 50, and especially, over 60 years of age [14].

Subanalysis by borough further demonstrated that certain areas in New York City bore the greater brunt of the pandemic. Boroughs such as the Bronx, Queens and Brooklyn had higher average YPLL per person at 16.00, 15.55 and 15.00, respectively. Further, the total VSL of the Bronx, Queens and Brooklyn was nearly five-times that of Staten Island and double that of Manhattan. Between Staten Island and Manhattan, Staten Island had a higher average YPLL per person (14.47 vs 13.43), but a lower total VSL (US$4.89 vs 10.66 billion), possibly due to variation in population size, population density, demographics, social and environmental context, and patient-level health risks. Differences across boroughs are likely a reflection of the varying socioeconomic, racial and ethnic backgrounds. According to the US Census, the Bronx, Queens and Brooklyn are predominantly non-white and home to a higher percentage of African Americans, Asians and Hispanic/Latino residents compared with Staten Island and Manhattan. These three boroughs also have a greater percentage of individuals living in poverty and without health insurance [15]. These differences are consistent with prior literature displaying associations between low-income neighborhoods, a predominantly non-white population, and limited access to healthcare and testing with high COVID-19 positivity rates in New York City [12].

Given the ongoing COVID-19 pandemic, one important limitation of our analysis is that these values may neither represent the latest data nor the full extent of the pandemic as new data are acquired and released daily. Thus, the calculated YPLL and VSL are likely an underestimation of the actual loss of life across New York State due to this pandemic. Another important limitation is the lack of data stratified by both age and other demographic characteristics such as race, ethnicity and socioeconomic status. This limits the scope of our analysis regarding the contributing factors behind the disparities in YPLL and VSL across the five boroughs. The estimated statistics are further limited by the lack of data stratified by both age group and sex. As a result, the average of males and female period life expectancy were used to calculate the midpoint in determining the YPLL. At last, the methodology of estimating VSL has its limitations [16–20]. There exist multiple methods for determining VSL and monetary values of VSL may differ based on national wealth and average income, as well as economic philosophy or school, among many other factors [17]. Moreover, the VSL utilized in this paper was derived from a set VSLY that assumed a standard value of life, regardless of age, income and race. Since COVID-19 disproportionally affects individuals of color, those of lower socioeconomic status and older age, it is important to consider these factors when determining a set VSLY.

## Conclusion

The estimated YPLL and statistical value of lives lost in New York State and New York City, since the beginning of the COVID-19 pandemic, are substantial. Among the five New York City boroughs, Queens and Brooklyn bore the greatest economic burden of lives lost, followed by the Bronx, Manhattan and Staten Island. These data may inform appropriate and effective policy strategies to help New York State and New York City manage the ongoing COVID-19 pandemic, limit further economic losses and loss of life, and prepare for a postpandemic recovery.

**Summary points**

- The estimated years of potential life lost and statistical value of lives lost in New York State and New York City, since the beginning of the COVID-19 pandemic, are substantial.
- As of 8 March 2021, the economic burden of lives lost was US$119.62 billion in New York State, with New York City accounting for an overwhelming majority of the cost at US$90.45 billion.
- State- and city-level analysis demonstrates that the economic burden of COVID-19 was predominantly concentrated in older age groups.
- Among the five New York City boroughs, Queens and Brooklyn bore the greatest economic burden of lives lost, followed by the Bronx, Manhattan and Staten Island.
